# Supplementary material for: Eleutheroside E supplementation prevents radiation-induced cognitive impairment and activates PKA signaling via gut microbiota
Source: Commun Biol. 2022 Jul 8;5:680. doi: 10.1038/s42003-022-03602-7 (PMC9270490; doi:10.1038/s42003-022-03602-7)
Supplement: Supplementary file 2 — Supplementary Information [file 42003_2022_3602_MOESM2_ESM.pdf]

Supplementary Table 1

Supplementary Table 1 The PCR primer sequences

Genes	Primer sequences（5’-3’）
β-actinF	ATCACTATTGGCAACGAGCGGTTC
β-actinR	CAGCACTGTGTTGGCATAGAGGTC
TNF-αF	GTCCGGGCAGGTCTACTTTG
TNF-αR	GGGGCTCTGAGGAGTAGACA
IL1-βF	TGCCACCTTTTGACAGTGATG
IL1-βR	ATGTGCTGCTGCGAGATTTG
IL-6	TGATGGATGCTACCAAACTGGA
IL-6	TGTGACTCCAGCTTATCTCTTGG
occludinF	GTCCTCCTGGCTCAGTTGAA
occludinR	AGAGTACGCTGGCTGAGAGA
claudin-3F	ACTGCGTACAAGACGAGACG
claudin-3R	TCCCTGATGATGGTGTTGGC
ZO-1F	TCTTGCAAAGTATCCCTTCTGT
ZO-1R	GAAATCGTGCTGATGTGCCA
