# Supplementary material for: Eleutheroside E supplementation prevents radiation-induced cognitive impairment and activates PKA signaling via gut microbiota
Source: Commun Biol. 2022 Jul 8;5:680. doi: 10.1038/s42003-022-03602-7 (PMC9270490; doi:10.1038/s42003-022-03602-7)
Supplement: Supplementary file 3 — Description of Additional Supplementary Files [file 42003_2022_3602_MOESM3_ESM.pdf]

Description of Additional Supplementary Files

File name: Supplementary Data 1
Description: source data behind the graphs in the paper.
